# Alignment of preferences in the treatment of multiple myeloma – a discrete choice experiment of patient, carer, physician, and nurse preferences

**DOI:** 10.1186/s12885-020-07018-6

**Published:** 2020-06-11

**Authors:** Simon J. Fifer, Kerrie-Anne Ho, Sean Lybrand, Laurie J. Axford, Steve Roach

**Affiliations:** 1 CaPPRe, Level 28, 161 Castlereagh St, Sydney, NSW 2000 Australia; 2 Present Address: Evidera, London, UK; 3 Amgen Australia, 123 Epping Rd, Macquarie Park, NSW 2113 Australia; 4 Myeloma Australia, 333 Swan St, Richmond, Victoria 3121 Australia

**Keywords:** Multiple myeloma, Patient preferences, Carer preferences, Physician preferences, Nurse preferences, Discrete choice experiment, Conjoint analysis

## Abstract

**Background:**

Multiple Myeloma (MM) is a cancer characterised by the proliferation of malignant plasma cells in the bone marrow. This study examined the treatment preferences of people living with MM compared to the treatment preferences of other groups involved in treatment decision making, including carers, as well as physicians and nurses who treat people living with MM in Australia.

**Methods:**

Data were collected using discrete choice experiments (DCEs) through an online survey. The DCEs presented participants with a traditional treatment generic choice experiment (e.g., treatment A vs treatment B), focusing on the clinical benefits of treatments and the associated risks. The attributes and levels of the attributes were selected based on previous research, literature review, qualitative research and expert opinion. The DCE data were modelled using a Latent Class Model (LCM).

**Results:**

The model revealed significant heterogeneity in preferences for treatment attributes. In particular, overall survival, remission period and annual out of pocket cost were the attributes with the most variation. In comparison to people living with MM, carers were less cost-sensitive and more concerned with quality of life (remission period). Physicians and nurses were generally more concerned with overall survival and more cost sensitive than people living with MM.

**Conclusions:**

This study demonstrated that not all people living with MM valued the same treatment attributes equally. Further, not all groups involved in MM treatment decision making had preference alignment on all treatment attributes. This has important implications for healthcare policy decisions and shared decision making. Results from this study could be used to guide decisions around the value of new MM medicines or the medical plan surrounding the needs of those living with MM, as well as those caring for them.

## Background

Multiple Myeloma (MM) is a disorder of the plasma cells characterised by the proliferation of malignant plasma cells in the bone marrow [1]. Worldwide incident cases of MM from 1990 to 2016 have increased by 126% [2]. While there is no cure for MM, recent advances in the understanding of the disease have resulted in new treatment options [3, 4] and subsequently, greater survival outcomes for people living with MM [4, 5]. This has led to a paradigm shift where MM is now considered a chronic condition, with people more likely to use multiple combinations of treatments, over longer periods of time.

At present, MM can be treated with several classes of treatments including immunomodulators, proteasome inhibitors, monoclonal antibodies, and others [3, 5]. These treatments differ with respect to clinical outcomes such as the average length of survival, potential side effects, as well as non-clinical aspects such as the mode and frequency of administration, and whether it is used as a monotherapy or combination therapy.

It is important to understand the treatment preferences of people living with MM, as well as of those who are actively involved in their care, such as carers, physicians and nurses. People living with MM are faced with a complex trade-off between benefits and risks across the abundance of clinically approved treatments available, within the broader complex of the condition and its impacts. People’s preferences for treatment are increasingly considered integral to the evaluation of new treatment options [6–8] and to the treatment and care process [9, 10]. Further, the alignment between preferences of people living with MM and of those involved in their care have been associated with improved treatment adherence and improved outcomes [9, 11]. There is a paucity of research examining the treatment preferences of people living with MM and that of those involved in their treatment and care. However, the research that has been conducted suggest that there may be differences between the preferences of people living with MM and that of physicians, such as the importance of quality of life [12, 13]. Further, physicians may also value other clinical indicators of disease, outcome and prognosis that people living with MM may not consider when evaluating a treatment [14].

Given the interaction and involvement of the care team with the patient in treating MM, this study aims to quantify treatment preferences and the alignment of treatment preferences for people living with MM and the care team (carers, physicians and nurses). Discrete choice experiments (DCEs) were used to empirically quantify preferences for treatment. DCEs have a firm theoretical basis in Random Utility Theory initially developed by Thurstone [15] and further developed by Mcfadden [16], combined with Lancaster’s theory of value [17]. Based on this framework, Louviere and Hensher [18] and Louviere and Woodworth [19] developed the empirical design approach to DCE.

DCEs are increasingly used to quantify patient and other stakeholder preferences for treatments in a broad range of diseases [20–22] and allow for an understanding of the underlying characteristics, or attributes, of treatment that influence patient choice. This is the first study to examine treatment preference alignment between people living with MM and the carers, physicians and nurses involved in their care and is clinically important in the care and management of people diagnosed with MM.

## Methods

An online survey with a DCE component was designed to understand the treatment preferences of people living with MM and that of their care team. The DCE comprised of choice tasks where participants were presented with different hypothetical treatment profiles and asked to select preferred treatment. Additional demographic questions were also included in the survey. The care-related quality of life scale (CarerQOL) [23] was also included in the carer survey to assess their quality of life. The CarerQoL consists of the CarerQoL-7D which measures subjective burden and the CarerQoL-VAS which measures wellbeing. Higher scores indicate less burden and higher wellbeing on the CarerQoL-7D and Carer-VAS respectively.

### Participants

One hundred twenty-four people living with MM, 44 carers, 28 haematologists, and 34 nurses who were involved in the care and treatment of people living with MM completed the DCE task. Participants were recruited through *Myeloma Australia*, a myeloma consumer advocacy group, and through specialist healthcare research panels.

### Study design

Qualitative interviews were conducted initially to obtain an awareness of the MM therapy area from patient and carers perspectives, the insights of which may assist in the ability to access new treatments for current and future patients. A total of 23 in-depth interviews each lasting between 45 and 60 min were conducted. Twelve interviews were conducted with patients (seven face to face; five telephone) and seven interviews were conducted with MM carers (three face to face; four telephone). Four interviews were conducted with haematologists (two interviews: one face to face; one telephone) and nurses with a special interest in Multiple Myeloma (two telephone interviews). Qualitative interviews provided a deeper understanding of the experiences and challenges in the treatment of MM as well as the attributes that contributed to treatment decision-making and determined the appropriate terminology for each respondent group. The results of the qualitative research were critical to inform the design of the DCE.

The design of the DCE, including the selection of attributes (i.e., treatment characteristics) and levels for inclusion, were informed by existing literature, qualitative in-depth interviews, discussions with patient advocacy groups and expert opinion. After reviewing the importance of attributes and levels to each subgroup, the following were included in the DCE: mode & frequency of administration [24], annual out of pocket costs, average overall survival [12, 14], remission period [25] and side-effects [12, 14, 26]. In addition to the chosen attributes, further therapies [13] was initially considered but not included in the DCE. Given the links between certain attributes and the cognitive limitations of respondents’ abilities to accurately evaluate a large number of attributes, a nested design combining multiple attributes was used. Table 1 lists the four treatment attributes and the levels associated as determined by the study team.

**Table 1:** Attributes and levels used for the DCE

Attribute	Level used in design		Level used in model^b^	
Average overall survival^a^ & Remission period	1 year survival	3 months remission	1	Short
		9 months remission	1	Long
	3 years survival	9 months remission	3	Short
		2 years 3 months remission	3	Long
	5 years survival	1 year 3 months remission	5	Short
		3 years 9 months remission	5	Long
	7 years survival	1 year 9 months remission	7	Short
		5 years 3 months remission	7	Long
	9 years survival	2 years 3 months remission	9	Short
		6 years 9 months remission	9	Long
Side effects	Mild or moderate	None	0	
		20% risk, lasting up to 2 months	20	
		20% risk, lasting longer than 2 months	20	
		40% risk, lasting up to 2 months	40	
		40% risk, lasting longer than 2 months	40	
		60% risk, lasting up to 2 months	60	
		60% risk, lasting longer than 2 months	60	
	Severe	None	None	
		5% risk, lasting up to 2 months	Some	
		5% risk, lasting longer than 2 months	Some	
		10% risk, lasting up to 2 months	Some	
		10% risk, lasting longer than 2 months	Some	
Mode & Frequency of administration	Oral	Daily	Oral	
		Weekly	Oral	
	Subcutaneous (SC)	2–3 times per week	SC - high	
		Weekly	SC - high	
		Fortnightly	SC - low	
		Monthly	SC - low	
	Intravenous (IV)	2–3 times per week	IV - high	
		Weekly	IV - high	
		Fortnightly	IV – low	
		Monthly	IV - low	
Annual out of pocket costs^c^	$0		0	
	$500		5	
	$1500		15	
	$3000		30	
	$5000		50	

^a^Average overall survival was treated as a quantitative variable in the model

^b^includes regrouping of levels for certain attributes

^c^Divided by 100 for modelling purposes

An unlabelled design was used, where the treatment profiles were displayed as ‘Treatment A’ and ‘Treatment B’. The experimental design followed good practice guidelines [27] and the combinations of levels presented in the tasks were designed using a D-efficient design structure based on naïve priors accounting for parameter sign [28] in Ngene. The final design included 10 scenarios in each of the 7 blocks,which were randomly distributed among the target sample. The following attributes were nested in the design because they are linked; Mode / frequency of treatment, average survival / remission period and risk / duration of side-effects.

Participants were presented with 10 choice tasks where they were presented with two hypothetical treatment profiles to select from and the option to opt-out. The opt out or neither treatment option would mean that respondents have no preference for Treatment A or Treatment B and would be indifferent to the treatments presented. The choice question was framed differently to allow each subgroup to think about the treatment decision as given in Table 2.

**Table 2:** Framing of choice question

Sub-group	Choice question
Patients	(If received prior treatment): For each scenario please imagine that your doctor has advised that you need to receive further treatment and you have a choice between the given options. You can choose treatment A, treatment B or you can choose ‘neither’ if you have no clear preference for either.
	(If not received prior treatment): For each scenario please imagine that your doctor has advised that you need to undergo treatment and you have a choice between the given options. You can choose treatment A, treatment B or you can choose ‘neither’ if you have no clear preference for either.
Nurses	For each scenario please imagine that the doctor has advised your patient that they need to receive treatment and they have a choice between the given options. Patients can choose treatment A, treatment B or they can choose ‘neither’ if they have no clear preference for either. Please indicate which choice you think would be your preference for the patient.
Physicians	For each scenario please imagine that you have advised your patient that they need to receive treatment and they have a choice between the given options. Patients could choose treatment A, treatment B or they could choose ‘neither’ if they have no clear preference for either. Please indicate which choice would be your preference for the patient.
Carers	For each scenario please imagine that the doctor has advised that the person you care for will need to receive treatment and you have a choice between the given options. You can choose treatment A, treatment B or you can choose ‘neither’ if you have no clear preference for either.

**Fig. 1:**
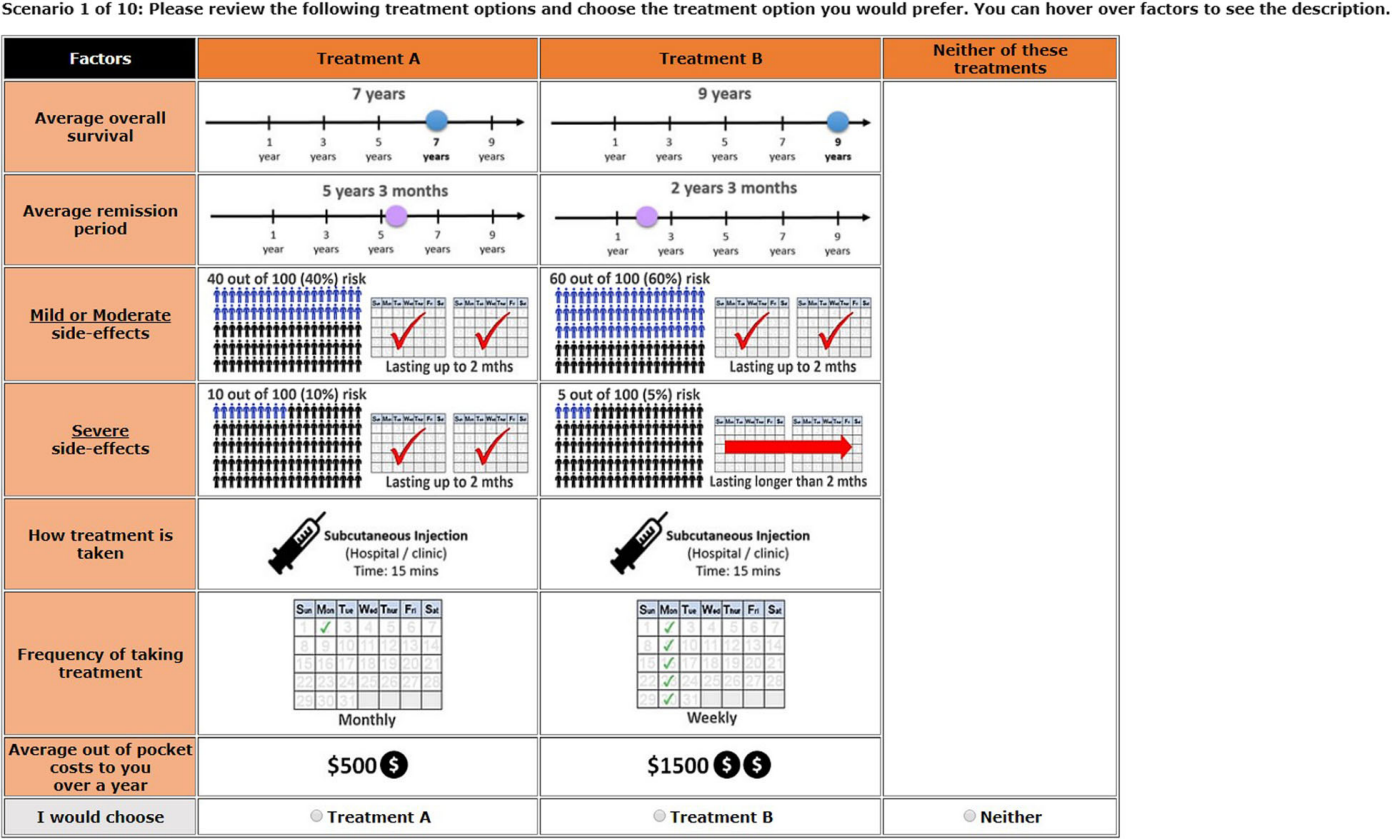
Example of a DCE choice task

Each treatment profile displayed represented a different combination of the attribute levels displayed in Table 1. An example of a choice task displayed to patients is shown in Fig. 1.^1^

### Analysis

Data from one person living with MM who incorrectly completed the carer survey were excluded from analysis. Descriptive statistics were used to examine the demographic and treatment characteristics of the participants. The DCE data for each subgroup were stacked and pooled and analysed using a latent class model (LCM). Setting up the data in this way allows some attributes to be treated as the same across the groups and some attributes allowed to vary (i.e., differ between the groups). Additionally, combining the four different respondent samples (patients, carers, physicians and nurses) allowed for more powerful analysis of a larger sample of 219 participants.^2^

LCMs allow for preference heterogeneity by allowing utility parameters to vary via discrete distributions (i.e., segments of respondents can have different sensitivities or parameter weights for each of the features) [29]. The LCM can also be used to understand different preference profiles of participants by identifying the influence of participant characteristics on treatment preferences through a class assignment model. Models were specified and estimated in Nlogit version 6 (Econometric Software, Inc.) and *p* < .05 criteria was used to determine statistical significance.

## Results

### Participant demographics

Demographics for 124 people living with MM (*N* = 53 females), 43 carers (*N* = 30 females), 28 physicians (*N* = 1 female) and 34 nurses (*N* = 30 females) are displayed in full in Table 3.

**Table 3:** Participant demographic characteristics

Demographic characteristics		People living with MM (*N* = 124)	Carers (*N* = 43)	Physicians (*N* = 28)	Nurses (*N* = 34)
Gender *N* (%)	Male	71 (57.26)	13 (30.23)	25 (89.29)	4 (11.77)
	Female	53 (42.74)	30 (69.77)	1 (3.57)	30 (88.24)
	Prefer not to answer			2 (7.14)	
Age *N* (%)	18–30	1 (0.80)			5 (14.71)
	31–40	1 (0.80)	1 (2.33)	9 (32.14)	10 (29.41)
	41–50	7 (5.65)	5 (11.63)	12 (42.86)	8 (23.53)
	51–60	32 (25.81)	19 (44.19)	6 (21.43)	10 (29.41)
	61–70	53 (42.74)	9 (20.93)	1 (3.57)	1 (2.94)
	71–80	27 (21.77)	9 (20.93)		
	81 or older	3 (2.42)			
Employment status *N* (%)	Working (full-time)	20 (16.13)	11 (25.58)	24 (85.71)	18 (52.94)
	Working (part-time/casual)	9 (7.26)	11(25.58)	3 (10.71)	16 (47.06)
	Not working	13 (10.48)	5 (11.63)		
	Retired	79 (63.71)	14 (32.56)		
	Other/prefer not to answer			1 (3.57)	
Location *N* (%)	Metro/City	78 (26.90)	29 (67.44)	23 (82.14)	26 (76.47)
	Regional	30 (24.19)	9 (20.93)	4 (14.29)	7 (20.59)
	Rural	16 (12.90)	5 (11.63)	1 (3.57)	1 (2.94)
Living Situation *N* (%)	Couple family with no children	65 (52.42)			
	Couple family with children	33 (26.61)			
	One parent family	3 (2.42)			
	Single person household	14 (11.29)			
	Group household (i.e., shared)	5 (4.03)			
	Other	4 (3.23)			
Cares for dependent family members *N* (%)		28 (22.58)			
Type of carer *N* (%)	Informal		42 (97.67)		
	Formal		1 (2.33)		
Relationship (of informal carer) to patient *N* (%)	Spouse		41 (95.35)		
	Other relative		2 (4.65)		
Years of experience treating people with MM *N* (%)	2 years or less				3 (8.82)
	3–6 years			4 (14.29)	12 (35.29)
	7–10 years			11 (39.29)	5 (14.71)
	Over 10 years			13 (46.43)	14 (41.18)
Number of people with MM seen in a month *M* (*SD*)				13.21 (7.49)	12.38 (8.84)
Place of practice *N* (%)^a^	Public hospital			22 (78.57)	22 (64.71)
	Private hospital			4 (14.29)	7 (20.59)
	Outpatient clinic			3 (10.71)	3 (8.82)
	Private practice			7 (25.00)	5 (14.71)

*N* – sample size, % – percentage, M – mean, *SD* – standard deviation, ^a^Places of practice are not mutually exclusive, total does not sum to 100%

The median length of time since people’s MM diagnosis was 4.34 years (SD = 5.52 years) and their most recent line of treatment lasted on average 1.24 years (*SD* = 1.73). Overall, 87.90% of people living with MM who completed the survey were currently receiving treatment or had received treatment for MM in the past. People’s current treatment situations are displayed in Table 4. On average, carers rated their subjective burden 9.72 (*SD* = 2.54) on the CarerQoL-7D and their wellbeing 6.81 (*SD* = 2.29) on the CarerQoL-VAS.

**Table 4:** Treatment characteristics of people living with MM

Current treatment situation	*N* (%)
Have been diagnosed with myeloma but don’t require treatment	2 (1.61)
Require first treatment but have not started yet	1 (0.81)
Myeloma has returned and need to start treatment again soon	6 (4.84)
In remission following treatment	43 (34.68)
Currently receiving treatment	60 (48.39)
Other	10 (8.07)
Not sure	2 (1.61)

*N* – sample size, % - percentage

### Latent class model

The median time for survey completion was 23.11 min. Participants indicated that they had a good level of understanding of the task, and that the task was easy to complete, with an average understanding of 8.44 (*SD* = 1.84), and an average ease rating of 7.34 (*SD* = 2.33) on a 10-point scale. In particular, self-reported average understanding for care teams were 8.21 (SD = 2.16) for carers, 9.21 (SD = 1.11) for nurses, and 8.46 (SD = 1.46) for physicians. Further average ease ratings were 7.00 (SD = 2.34) for carers, 7.64 (SD = 2.33) for nurses, and 7.71 (SD =1.56) for physicians.

The nested attributes and levels used in the design were rolled back and regrouped to facilitate modelling and interpretation. For example, daily and weekly oral treatments were regrouped and modelled as *high frequency* whereas subcutaneous fortnightly and monthly treatments were regrouped as *low frequency* in the analysis. Table 1 shows how nested attributes and levels were regrouped for model estimation. As an unlabelled experiment, all parameters were specified as generic across alternatives in the utility function.

The best fitting model was an LCM with two latent classes, based on AIC and BIC criteria (Table 5). The overall goodness of fit was assessed using the adjusted McFadden Pseudo R-squared, which takes into account the number of parameters in a model. The model fit results illustrate that the model provides a superior fit to a constant only model (*p* < .05). The average class probabilities were 0.61 and 0.39 for class 1 and class 2 respectively. More sophisticated models were also conducted to examine preference heterogeneity, including a Mixed Multinomial Logit Model (MMNL). These models were used as a guide to explore heterogeneity; however, the LCM was ultimately chosen as the preferred model as it fits the data well and didn’t require distributional assumptions using random parameters. The models identified significant heterogeneity in overall survival, remission period and average cost parameters between groups (*p* < .05). The final model presented takes this into account by using generic parameters where there was no heterogeneity between groups and using interaction terms where there was significant heterogeneity between groups.

**Table 5:** LCM output parameters

	Class 1		Class 2	
Class Proportions	0.61		0.39	
Utility parameters	Parameter	T-Ratio	Parameter	T-ratio
Generic parameters				
Subcutaneous - high frequency *(Reference category: oral)*	−0.899*	−3.380	− 0.802*	−4.800
Subcutaneous - low frequency *(Reference category: oral)*	−0.899*	−3.380	0	0
Intravenous - high frequency *(Reference category: oral)*	−0.940*	−3.470	− 0.753*	− 4.380
Intravenous - low frequency *(Reference category: oral)*	− 0.940*	− 3.470	− 0.514*	− 3.110
Out of pocket cost	− 0.012*	−2.400	−0.017*	− 4.140
Overall survival	1.226*	8.670	0.291*	9.440
Short remission period *(Reference category – long remission period)*	−1.059*	−5.860	−0.750*	−5.440
Mild side effects	−0.009*	−2.230	−0.009*	− 2.890
No severe side effects *(Reference category – some severe side effects)*	1.251*	3.930	0.644*	3.960
Neither treatment constant	−2.294*	−3.390	−0.710*	− 3.020
Interaction effect parameters				
Out of pocket costs (Carers) *(Reference category – out of pocket costs – people living with MM)*	0	0	0	0
Out of pocket costs (Physicians) *(Reference category – out of pocket costs – people living with MM)*	0	0	−0.033*	−2.240
Out of pocket costs (Nurses) *(Reference category – out of pocket costs – people living with MM)*	0	0	−0.064*	−3.780
Overall survival (Carers)	0	0	0.108*	2.490
Overall survival (Physicians)	0	0	0.449*	4.120
Overall survival (Nurses)	−0.468*	−2.970	0.439*	4.250
Short remission period (Carers) *(Reference category – long remission period – people living with MM)*	−1.366*	−2.770	0	0

*p < .05, Restricted log likelihood: − 2405.961, Log likelihood: − 1022.241, Rho-squared: 0.452, Number of respondents: 219, Number of choice observations: 2190

The aim of using interaction effects in the LCM was to show how preferences differ across respondents’ subgroup (patients, carers, physicians & nurses). Alternative approaches were considered to address differences between groups such as inclusion in the class membership function and separate models for each group. However, given the sample size limitations, it was decided that the use of interaction effect is the most appropriate method for assessing these differences across samples.

Overall, the attributes that were important and the direction of the preferences were similar for Class 1 and Class 2, though there were differences in the magnitude of the preference. Both classes were associated with a greater preference for oral treatments, lower out of pocket costs, longer periods of overall survival, longer remission periods, lower risk of mild side effects and no severe side effects (*p* < .05). All else held constant, the two classes also preferred choosing a new treatment compared to the neither treatment constant (*p* < .05), though the magnitude of this preference was greater for Class 1.

There was also heterogeneity within the classes between people living with MM and their care team. For Class 1, longer periods of overall survival were significantly more important to people living with MM than nurses (*p* < .05) whereas longer remission periods were significantly more important to carers than people living with MM (*p* < .05).

For Class 2 as indicated by the interaction effect parameters in Table 5, lower out of pocket costs were significantly more important to physicians and nurses than people living with MM (*p* < .05). Further, longer periods of overall survival were significantly more important to physicians, nurses and carers than people living with MM (*p* < .05).

Parameter restrictions were imposed where necessary to improve identifiability of the parameters and facilitate comparisons between classes. Equality constraints were imposed for parameters which were found to have close parameter estimates. For example, equality constraints were imposed between high and low frequency attributes for Subcutaneous and Intravenous treatment modes in class 1. With no restrictions imposed, the above parameter estimates were very close and showed minimal difference between high and low frequency. This implies that respondents’ choice is driven by mode rather than frequency. Further, fixed/zero value restrictions were imposed on a number of parameters in class 1 and class 2 which were not significant (Table 5).

#### Attribute importance

**Fig. 2:**
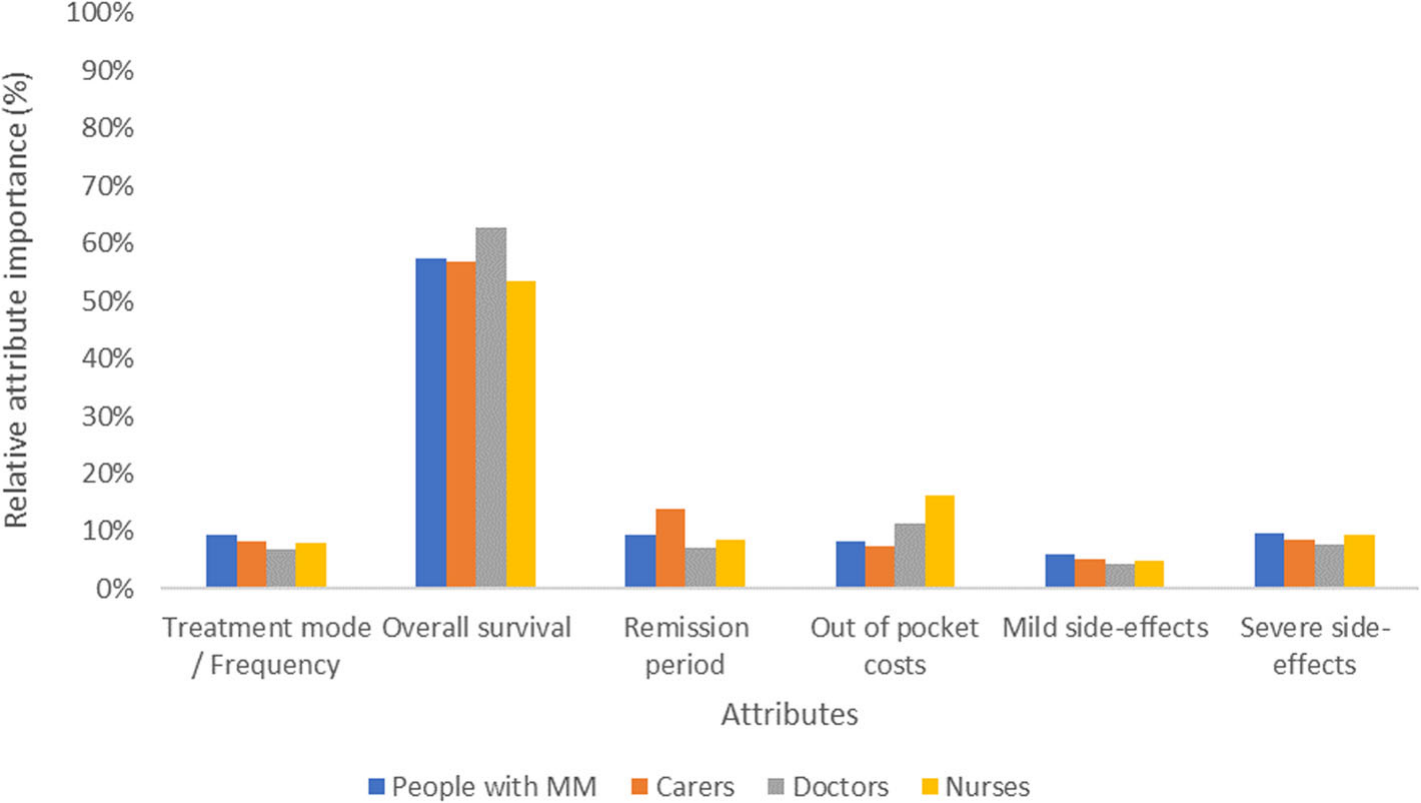
Attribute importance by group. *Note: Please refer* Table 1*for details on attributes and levels and* Table 5*for parameters used to calculate attribute importance.*

In standard choice models, the relative attribute importance cannot be compared directly using attribute parameter size and significance. This is because the attributes represented by each parameter are presented on different scales. However, the model can be used to evaluate the importance of attributes by comparing the utility derived from the lowest to the highest level for each attribute [30]. The resulting change in utility for each attribute can be compared and used to calculate the relative attribute importance. Figure 2 presents the relative importance of each of the attributes (treatment mode/frequency, overall survival, remission, out of pocket costs, mild sild effects, and severe side effects), by group. Attribute importance is considered relative because it is based on attribute levels chosen for this experiment. Attribute importance results should be viewed within this context.

Not surprisingly, overall survival was considered the most important attribute across all participant groups. However, physicians placed more importance on overall survival than other groups. In contrast, the risk of mild-side effects was considered the least important attribute across all participant groups. There was also disparity between physicians/nurses and people living with MM/carers in the importance of out of pocket costs. The length of remission was valued differently between the different groups, with carers considering remission more important than the people living with MM, physicians, and nurses.

## Discussion

This is the first study to examine the alignment of treatment preferences between people living with MM and that of their carers, physicians, and nurses. Overall, people living with MM and their care team had similar preferences for treatment attributes, valuing clinical as well as non-clinical treatment attributes. As expected, clinical treatment attributes of importance include length of overall survival and remission and the risk of side effects. Interestingly, non-clinical treatment attributes of importance include mode of administration, with a preference for oral treatments and lower out of pocket costs. It is clear that the preference for oral treatments reflects a preference for oral modes of treatment administrations over injection and infusion modes of treatment. However, in the current study and preferences of the sample group, the preference for oral treatments may also indicate a preference for other convenience factors as oral treatments are self-administered and do not involve travel to a hospital or clinic. It is unclear whether this finding of treatment mode holds in other countries where injections may be administered at home. Injectables are an important component to the MM treatment regimen, and the multifactorial nature of MM treatment across the disease course means that injectables are currently an inevitable therapeutic option.

Results demonstrate that people living with MM and their care team can further be separated into two classes (or groups) demonstrating that not all participants have the same treatment preferences. The two classes have similar treatment preferences for treatment attributes but with different magnitudes of value, demonstrating that people are not homogenous in their treatment preferences. For example, longer overall survival and remission was more important to participants who belong to Class 1 than those who belong to Class 2.

Across the two classes, there were also differences in treatment preferences between people living with MM and their care team. Consistent with previous research into the preference of people living with MM and physicians [13], the present findings demonstrate that while the preferences of people living with MM and their care team do mostly align, there is also heterogeneity in the magnitude of utility for some attributes. Interestingly, overall survival is relatively more important to physicians and nurses than people living with MM and carers. This is consistent with the findings from Muhlbacher and Nubling [13] that show prolonged life expectancy is more important for physicians relative to people living with MM. Remission period is also more important to carers than the other decision-makers. Further, carers are also less concerned with out of pocket treatment costs than people living with MM. Given that carers are typically a spouse or other family member, it is speculated that carers’ treatment preferences are driven by the desire for optimal long-term outcomes and quality of life (for themselves and their spouse/family member). It is also speculated that carers may value remission periods more highly as it has a personal component – it represents a period of less intense care requirements, which may have important mental, physical and economic aspects that were not further investigated by this research study. Interestingly, in this study out of pocket costs is more important to physicians and nurses than people living with MM and carers, suggesting that physicians and nurses overestimate the importance of out of pocket costs for people living with MM in the treatment decision process.

Overall, the findings demonstrate that while clinical treatment attributes are important, people living with MM and their care team also consider non-clinical treatment attributes to be important when making treatment decisions. This provides key information beyond what can be examined in clinical trials and has important practical implications for people living with MM and their care team in discussions about treatment choices. Previous research shows that patient-physician alignment in treatment preferences can lead to improved treatment adherence and subsequently improved treatment outcomes [9, 11]. Given the role of carers and nurses in the care and treatment of people living with MM, it is anticipated that understanding the similarities and differences in treatment preferences between people living with MM and their care team will serve to further aid shared discussions around treatment decisions and medical plans surrounding the needs of those living with MM and those caring for them as well as treatment outcomes.

There are some limitations to the study that are mainly related to sampling. Firstly, the carers, physicians and nurses included in the study are not matched to people living with MM. Therefore, the similarities and differences between people living with MM and their care team can only be discussed at an aggregate level and not at an individual level. Further, the DCE task can only account for the general treatment preferences of physicians and nurses and cannot account for the possibility of different treatment preferences based on the individual circumstances of people living with MM and their carers.

Given these study limitations and the continued progression towards patient-centricity, it would of be interest for future research to examine the alignment of treatment preferences within matched groups of people living with MM and their respective care team. Further research may also focus on the deeper mental, physical and economic impacts on carers of those living with MM. Clinically and practically, it would also be of interest to use DCE tasks to aid people living with MM and their care team in shared-decision making discussions. Further, future research needs to delve in to examining the influence of demographic characteristics on treatment preferences for people living with MM and their respective care team.

## Conclusions

Overall, the study demonstrates that clinical as well as non-clinical treatment attributes are important to people living with MM and their care team. While there is some alignment between people living with MM and their care team in the treatment attributes they value, there is also some heterogeneity with regards to the magnitude of importance placed on each of the treatment attributes. These findings have important practical implications around understanding the needs and wants of people living with MM and their carers as well as shared-decision making and ultimately treatment adherence and outcomes.

## Data Availability

The datasets generated and/or analysed during the current study are not publicly available as they are owned by the pharmaceutical companies who funded the study.

## Abbreviations

CarerQOL: Care-related Quality of Life scale
DCE: Discrete Choice Experiment
LCM: Latent Class Model
MM: Multiple Myeloma
MMNL: Mixed Multinomial Logit Model

## Footnotes

1 Note that the description relevant to cost attribute presented in Figure 1 was presented differently for each respondent subgroup. For patients, it was “average out of pocket cost to you over a year” whereas for other groups “average out of pocket cost to the patient over a year”.

2 Other than patients, data was not sufficient to estimate each subgroup separately.
